# Functional perturbation of classical natural killer and innate lymphoid cells in the oral mucosa during SIV infection

**DOI:** 10.3389/fimmu.2012.00417

**Published:** 2013-01-08

**Authors:** Haiying Li, R. Keith Reeves

**Affiliations:** Division of Immunology, New England Primate Research Center, Harvard Medical School, One Pine Hill DriveSouthborough, MA, USA

**Keywords:** natural killer cells, innate lymphoid cells, oral mucosa, HIV, SIV

## Abstract

Despite the fact that the majority of human pathogens are transmitted across mucosal surfaces, including the oral mucosae, oral immunity is poorly understood. Furthermore, because the normal flora of the oral cavity is vast and significantly diverse, host immunity must balance a complex system of tolerance and pathogen recognition. Due to the rapid recognition and response to pathogens, the innate immune system, including natural killer (NK) cells, likely plays a critical role in mediating this balance. Because logistical and ethical restraints limit access to significant quantities of human mucosal tissues, non-human primate models offer one of the best opportunities to study mucosal NK cells. In this study we have identified both classical NK cells, as well as innate lymphoid cells (ILCs) in tonsillar and buccal tissues and oral-draining lymph nodes. Identified by mutually exclusive expression of NKG2A and NKp44, NK cells, and ILCs in the oral mucosa are generally phenotypically and functionally analogous to their gut counterparts. NKG2A^+^ NK cells were more cytotoxic while NKp44^+^ ILCs produced copious amounts of IL-17 and TNF-α. However, in contrast to gut, oral NK cells and ILCs both produced large quantities of IFN-γ and the beta-chemokine, MIP-1β. Also in contrast to what we have previously found in gut tissues of SIV-infected macaques, we found no reduction in NK cells during chronic SIV infection, but rather an expansion of ILCs in oral-draining lymph nodes and tonsils. These data suggest that the lentivirus-induced depletion of the NK cell/ILC compartment in the gut may be absent in the oral mucosa, but the inherent differences and SIV-induced alterations are likely to have significant impact on preventing oral opportunistic infections in lentiviral disease. Furthermore, these data extend our understanding of the oral innate immune system in general and could aid future studies evaluating the regulation of both normal oral flora and limiting transmission of oral mucosal pathogens.

## Introduction

Although natural killer (NK) cell research in humans and mice has focused heavily on blood and secondary lymphoid organs, more recent reports have provided convincing evidence for a diverse NK cell repertoire in the gastrointestinal (GI) and female reproductive tracts. Mucosal NK cells not only have prototypical cytolytic functions where they are among the first cells to intersect pathogens and eliminate neoplastic cells, but are also important for tissue remodeling and homeostasis (Pang et al., 1993; Cooper et al., 2001; Caligiuri, 2008; El Costa et al., 2008; Lanier, 2008; Tiemessen et al., 2009). By comparison, little is known about NK cells in distal mucosal tissues, such as the oral mucosa. Bulk NK cells in the labial and oropharyngeal mucosae are dense, constituting up to 40% of total lymphocytes and are primarily located submucosally and in the lamina propria, as indicated by immunohistochemistry (de Repentigny et al., 2004; Challacombe and Naglik, 2006; Zancope et al., 2010). However, cytotoxic NK cells have been found to migrate into epithelial spaces during periods of inflammation such as gingivitis or other periodontal disease (Komiyama et al., 1988; Challacombe and Naglik, 2006; Stelin et al., 2009). NK cells have also been shown to accumulate both intratumorally and peritumorally during oral cavity and lip squamous cell carcinomas (Zancope et al., 2010).

Recently, a subpopulation of mucosa-restricted cells that bear features similar to both NK cells and TH17 and TH22 has been identified in mice, humans, and rhesus macaques (Cella et al., 2009, 2010; Hughes et al., 2009; Reeves et al., 2011; Wills-Karp and Finkelman, 2011). These cells share a phylogenetic lineage with lymphoid tissue-inducing cells (LTis), express relatively high levels of NKp44^+^ and for the purpose of this report will be collectively referred to as NKp44^+^ innate lymphoid cells (ILCs) (Di Santo et al., 2010; Wills-Karp and Finkelman, 2011). ILCs are non-cytotoxic under normal conditions and may also have a role in maintaining epithelial homeostasis due to secretion of IL-17 and IL-22 (Cella et al., 2010; Di Santo et al., 2010). Unfortunately, due to limited access to mucosal tissues in humans, these cells have been problematic to study. We have recently identified and characterized these cells in the GI mucosae and mucosal-draining lymph nodes of rhesus macaques using 12-color flow cytometry (Reeves et al., 2011). It is, however, unclear if functional ILCs exist in other mucosal sites, including the oral mucosa and associated draining lymph nodes.

Evidence suggests that oral HIV infection is relatively common, and occurs most frequently through breast milk transmission by untreated mothers. Furthermore, although oral-genital HIV transmissions are generally considered to be rare, research suggests these infections can still occur (Campo et al., 2006). Although the mechanisms remain unclear, dendritic cells and macrophages are highly prevalent throughout the oral mucosae, and some evidence suggests these cells may capture HIV virions and transfer them to CD4^+^ T cells in the local lamina propria (Cutler and Jotwani, 2006). In healthy gingiva, the HIV receptors and co-receptors CD4, CCR5, CXCR4 are expressed at relatively low levels, but increase during periodontal disease (Cutler and Jotwani, 2006; Kweon, 2011). This suggests, similar to what occurs in the genital mucosa, secondary infections may increase oral transmission. Furthermore, oral epithelial cells may also transfer virus to CD4^+^ T cells in a mechanism involving alpha-galactosylceramide (Challacombe and Naglik, 2006; Moutsopoulos et al., 2006). Regardless of the mechanisms involved, oral HIV transmission occurs at lower rates than other routes of infection and may be abrogated by high concentrations of salivary enzymes that can disrupt virions as well as innate beta-chemokines that can block infection (Moutsopoulos et al., 2006; Lu and Jacobson, 2007).

NK cells have long been shown to inhibit HIV replication in *in vitro* cultures through both direct and indirect mechanisms, and are associated with prevention of disease progression in both humans and simian immunodeficiency virus (SIV)-infected macaque models (Bandyopadhyay et al., 1990; Vowels et al., 1990; Jenkins et al., 1993; Fehniger et al., 1998; Shieh et al., 2001; Alter et al., 2005; O'Connor et al., 2007; Ward et al., 2007; Pereira et al., 2008; Alter and Altfeld, 2009; Bostik et al., 2009). Empirical evidence suggests that NK cells may also have a specific role in limiting oral transmission of lentiviruses, including expression of NK cell KIR2DL3 and its ligand HLA-C1 which are associated with protection against mother-to-child transmission (MTCT) (Paximadis et al., 2011). NK cells have also been shown to have potent anti-HIV responses to envelope proteins that prevent infant infection *in utero* (Tiemessen et al., 2009), and a similar mechanism could exist for breast milk transmission, supported by the fact that functional NK cells are even found in human breast milk (Jin et al., 2011). During experimental oral SIV infection, CXCL10, a known chemoattractant for NK cells via the CXCR3-CXCL10 axis (Milush et al., 2007; Durudas et al., 2011), is significantly upregulated in the oral mucosae and alimentary tract. Although recruitment of NK cells to oral tissues may occur too late to prevent transmission, this phenomenon could be exploited in future designs of immunotherapeutics and vaccines. Passive immunization of newborn rhesus macaques can prevent oral SIV transmission and protection is at least partially dependent on antibody-dependent cell-mediated virus inhibition (ADCVI) mediated by NK cells (Van Rompay et al., 1998; Forthal et al., 2006). Taken together, the fact that oral transmissions of HIV/SIV occur less frequently than other types of transmission, and potently functional NK cells are found in the oral mucosae and draining lymph nodes, supports the need for significant exploration in this area of research. In this study we sought to comprehensively characterize the biology of macaque NK cell and ILC subpopulations, their relative distribution in tissues outside peripheral blood, and ascertain the effects of chronic SIV infection on their phenotype and function.

## Materials and methods

### Animals and SIV infections

A total of 16 rhesus macaques (*Macaca mulatta*), all of Indian genetic background, were sampled for this study; 10 SIV-naïve and 6 macaques which had been initially intravenously infected with SIVmac239 (10 AID_50_) and then evaluated after a minimum of 12 months (chronic disease) for this study. All animals were housed at the New England Primate Research Center and maintained in accordance with the guidelines of the Committee on Animals of the Harvard Medical School and the Guide for the Care and Use of Laboratory Animals.

### Cell collection and processing

Processing of blood and tissue samples was carried out using assays optimized in our laboratory. Briefly, total peripheral blood mononuclear cells were isolated from EDTA-treated venous blood by density gradient centrifugation over lymphocyte separation media (MP Biomedicals, Solon, OH) and a hypotonic ammonium chloride solution was used to lyse contaminating red blood cells. Tonsils, buccal tissues, and mesenteric and oral lymph nodes (OLN) were all collected at scheduled necropsies. For biopsy collection, macaques were first anesthetized with ketamine HCL (10–20 mg/kg, IM) and 10–15 rectal biopsies were collected using 1.9 mm fenestrated endoscopic biopsy forceps (Olympus, Center Valley, PA). Tonsils and lymph nodes were dissected and lymphocytes were isolated using standard procedures in our laboratory (Reeves et al., 2010). Mononuclear cell isolation from buccal tissue and biopsy pieces were performed using mechanical and enzymatic disruption as described previously for mucosal specimens (Reeves et al., 2011).

### Polychromatic flow cytometry

Flow cytometry staining of mononuclear cells was carried out for cell-surface and intracellular molecules using standard protocols (Reeves et al., 2010). LIVE/DEAD Aqua dye (Invitrogen) and isotype-matched controls and/or fluorescence-minus-one (FMO) controls (Roederer, 2001) were included in all assays; a full list of antibodies is shown in Table 1. Acquisitions were made on an LSR II (BD Biosciences) and analyzed using FlowJo software (Tree Star Inc., Ashland, OR).

**Table 1:** **Antibodies used for flow cytometric analyses**.

**Antibody**	**Clone**	**Fluorochrome**	**Manufacturer**
anti-CD3	SP34.2	APC, APC-Cy7	BD Biosciences (La Jolla, CA)
anti-CD14	M5E2	PE-Cy7	BD Biosciences
anti-CD16	3G8	Alexa700, FITC	BD Biosciences
anti-CD20	L27	PerCp-Cy5.5	BD Biosciences
anti-CD45	D058-1283	FITC	BD Biosciences
anti-CD45	MB4-6D6	APC	Miltenyi Biotec (Gladbach, Germany)
anti-CD56	NCAM16.2	PE-Cy7	BD Biosciences
anti-HLA-DR	Immu-357	PE-Texas Red	Beckman-Coulter
anti-NKG2A	Z199	Pacific Blue^*^	Beckman-Coulter
anti-NKG2D	ON72	PE	Beckman-Coulter
anti-NKp44	Z231	PE, PerCp-Cy5.5	Beckman-Coulter
anti-NKp44	2.29	APC	Miltenyi Biotec
anti-NKp30	Z25	PE	Beckman-Coulter
anti-CD107a	H4A3	PerCp-Cy5	BD Biosciences
anti-IFN-γ	B27	APC	Invitrogen
anti-IL17a	eBio64DEC17	Alexa647	eBioscience
anti-MIP-1β	24006	FITC	R&D Systems
anti-TNF-α	Mab11	Alexa700	BD Biosciences

* In-house custom conjugate.

### Plasma viral load quantification

Total RNA copy number equivalents were determined in EDTA-treated plasma using a standardized quantitative real-time RT-PCR assay based on amplification of conserved *gag* sequences as described previously for this cohort of animals (Reeves et al., 2011).

### Intracellular cytokine staining assays

To assess NK cell function, we performed *ex vivo* functional analyses optimized in our laboratory using antibodies titered and tested to be cross-reactive in macaques. Briefly, 1 × 10^6^ mononuclear cells were resuspended in RPMI 1640 (Sigma-Aldrich) containing 10% FBS (R10) and either stimulated with PMA (50 ng/ml) and ionomycin (1 μg/ml) or cultured in medium alone. Anti-CD107a was added to each tube at a concentration of 20 μl/ml and Golgiplug (brefeldin A) and Golgistop (monensin) were added at final concentrations of 6 μg/ml, then all samples were cultured for 12 h at 37°C in 5% CO_2_. After culture, samples were surface-stained using markers to delineate live cells (LIVE/DEAD Aqua dye), leukocytes (CD45), and NK cell and ILC populations (CD3, NKG2A, NKp44). Cells were then permeabilized using Caltag Fix & Perm and intracellular cytokine staining was performed for combinations of MIP-1β, IFN-γ, TNF-α, and IL-17a. Unless otherwise stated reagents were purchased from BD Biosciences.

### Statistical analyses

All statistical and graphical analyses were performed using GraphPad Prism software (GraphPad Software, Inc., La Jolla, CA). Non-parametric Wilcoxon Matched Pairs, Mann–Whitney, and Spearman correlation tests were used where indicated and *P* < 0.05 were assumed to be significant.

## Results

### Oral mucosa contains both classical natural killer cells and innate lymphoid cells

**Figure 1:**
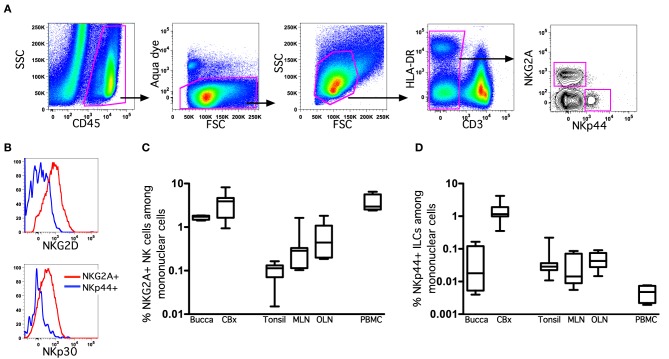
**Oral-associated mucosal tissues contain classical NK cells and innate lymphoid cells. (A)** Representative gating strategy to identify NKG2A^+^ NK cells and NKp44^+^ ILCs among live mononuclear cells. **(B)** Representative histograms of NKG2D and NKp30 expression on NK cells and ILCs. Frequencies of NK cells **(C)** and ILCs **(D)** among live CD45^+^CD3^−^ mononuclear cells in tissues of naïve and SIV-infected macaques are shown. Bar and whisker plots represent medians and ranges of 4–12 animals per group. CBx, colorectal biopsy; MLN, mesenteric lymph node; OLN, oral lymph node.

**Figure 2:**
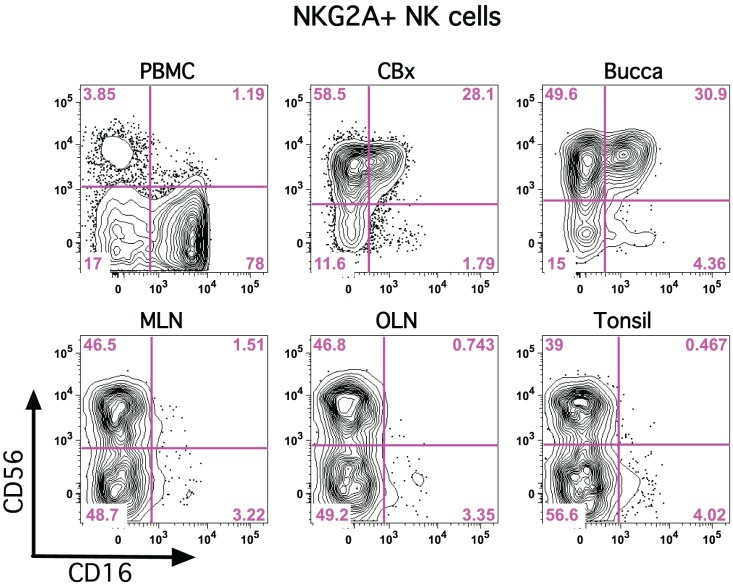
**CD56 and CD16 are disparately expressed on classical NK cells in tissues.** Representative flow cytometry plots are shown demonstrating expression of CD56 and CD16 on NKG2A^+^ NK cells in multiple tissues. PBMC, peripheral blood mononuclear cells; CBx, colorectal biopsy; MLN, mesenteric lymph node; OLN, oral lymph node.

To identify NK cell and ILC populations in oral mucosae and draining lymph nodes, we utilized polychromatic flow cytometry employing gating strategies previously developed specifically for rhesus macaques (Reeves et al., 2011). We first gated on CD45^+^ leukocytes to exclude any contaminating epithelial cells and excluded dead cells using LIVE/DEAD cell stain (Figure 1A). Among live CD45^+^CD3^−^ mononuclear cells we first identified classical NK cells as NKG2A^+^—NKG2A has been previously found to be the most useful NK cell inclusive marker for rhesus macaques and other Old World monkeys, despite its variability of expression in humans (Webster and Johnson, 2005; Pereira et al., 2008; Reeves et al., 2010). NKG2A^+^ NK cells were readily distinguished from ILCs that expressed high levels of NKp44, as previously described for GI tissues (Reeves et al., 2011). NK cells and ILCs also lacked CD20 and CD14 expression (data not shown). To further confirm the identity of oral NK cells, we also analyzed expression of NKG2D and NKp30, which were found at high levels on NKG2A^+^ NK cells, but were dim to negative on NKp44^+^ ILCs (Figure 1B). The frequency of NK cells and ILCs also varied widely in different oral tissues. (Figures 1C,D) To provide a frame of reference, we compared NK cells in oral tissues to another mucosal site, colorectum, as well as a distal mucosa-draining lymph node, mesenteric lymph nodes (MLN), and peripheral blood mononuclear cells (PBMC). Classical NKG2A^+^ NK cells were found at relatively similar frequencies in buccal and colorectal mucosae (median percentage among mononuclear cells, 1.7 and 3.8%, respectively). However, NK cells were ~10-fold less frequent in tonsils and OLN and MLN (median percentage among mononuclear cells, 0.11, 0.29, and 0.44%, respectively). Although in comparison to NK cells, NKp44^+^ ILCs are rare, they were by far most abundant in colorectal mucosa (median frequency, 1.1%), the tissue where we first described these cells in rhesus macaques. ILCs were detectable in tonsils and lymph nodes, albeit generally lower than 0.05% of mononuclear cells. As expected, NK cells were found at relatively high frequencies in blood, whereas NKp44^+^ ILCs were virtually absent. NK cells could be further delineated by expression of CD56 and CD16 into four subpopulations and distribution was highly disparate among tissues (Figure 2). As expected, peripheral blood was heavily dominated by CD16^+^ NK cells, whereas mucosal tissues were enriched for CD56^+^ NK cells. Although multiple studies have shown this phenomenon, this is the first report that the CD56^+^ enrichment is also present in buccal mucosa. OLN and tonsils were enriched for CD56^+^ and DN NK cells similar to distal lymph nodes sites, but generally lacked CD16^+^ NK cells.

### Functional profiles of NK cells and ILCs in the oral mucosa

**Figure 3:**
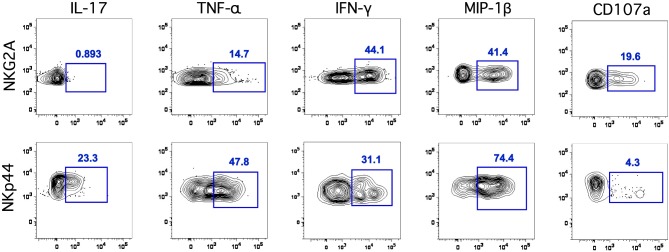
**Classical NK cells and ILCs in the oral mucosa have distinct functional profiles.** Representative flow cytometry plots depicting CD107a and intracellular cytokine responses in NK cells and ILCs in the oral mucosa following mitogen stimulation. ICS profiles are representative of a total of 13 animals.

We next evaluated the functionality of classical NKG2A^+^ NK cells and NKp44^+^ ILCs using a four-function ICS assay (Reeves et al., 2010). Due to limited quantities of cells isolated from oral tissues we only addressed functionality using OLN. Following mitogen stimulation NKG2A^+^ NK cells secreted no IL-17 and little TNF-α, but produced significant amounts of IFN-γ and MIP-1β (Figure 3). In contrast, ILCs secreted the regulatory cytokines IL-17 and TNF-α as well as IFN-γ and MIP-1β. The functional profile of ILCs in OLN was similar to that of GI ILCs with one notable exception—ILCs in the gut do not generally produce IFN-γ (Reeves et al., 2011). In response to stimulation, classical NK cells upregulated a surrogate marker for degranulation, CD107a, whereas ILCs expressed only very low levels. This indicated not surprisingly that ILCs were generally non-cytotoxic. Interestingly, both NK cells and ILCs were robust producers of MIP-1β, which could contribute to blocking of SIV infection and replication in the oral mucosa.

### Functional perturbation of oral NK cells and ILCs during SIV infection

**Figure 4:**
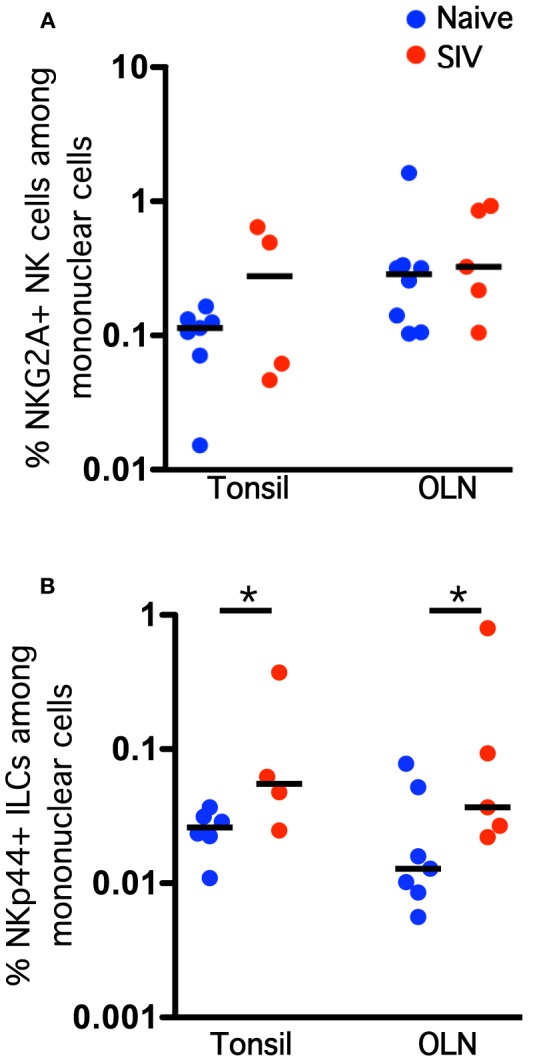
**Chronic SIV infection induces an expansion of NKp44^+^ ILC frequencies in the oral mucosa.** Frequencies of NKG2A^+^ NK cells **(A)** and NKp44^+^ ILCs **(B)** among live CD45^+^CD3^−^ mononuclear cells in oral tissues of naïve and SIV-infected macaques. Horizontal lines indicate medians of 4–8 animals per group. Mann–Whitney *U*-tests were used for naïve-vs.-SIV comparisons; ^*^P < 0.05.

**Figure 5:**
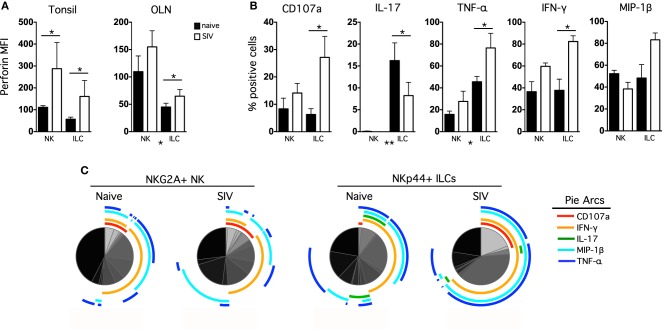
**Chronic SIV infection alters functional profiles of oral mucosal NK cells and ILCs. (A)** Intracellular perforin expression was determined in NK cells and ILCs *ex vivo*; bars represent means ± SEM for 6–8 animals per group. **(B)** OLN mononuclear cells were stimulated with PMA/ionomycin for 12 h and then CD107a expression, and IL-17, TNF-α, IFN-γ, and MIP-1β production were measured in NK cells and ILCs in naïve and SIV-infected macaques. The monofunctional profiles of each subpopulation were determined by expressing each response as a proportion of the total cell population. The means ± SEM for 6–8 animals per group are shown. **(C)** Multiparametric analyses were performed for each group using SPICE software (v.5.22). Mann–Whitney *U*-tests were used for naïve-vs.-SIV comparisons and Wilcoxon Matched Pairs tests were used to compare NK cells and ILCs; ^*^P < 0.05; ^**^P < 0.01. MFI, median fluorescence intensity; OLN, oral lymph nodes.

We know that NKG2A^+^ NK cells and NKp44^+^ ILCs play critical immune defense and homeostatic roles in the mucosa, and we have recently shown that lentivirus infection has a significant negative impact on both cell types (Reeves et al., 2011). Therefore, we next enumerated and evaluated functionally NK cells and ILCs in oral mucosal tissues of SIV-infected and naïve macaques. Although both classical NK cells and ILCs trended toward increase in tonsils and OLN of SIV-infected compared to naïve macaques, only the expansion of ILCs was significant (Figure 4). This finding was in stark contrast to our previous observations for other mucosal sites, such as the colorectal mucosa, where ILCs are massively depleted and classical NK cells are reduced (Reeves et al., 2011). Furthermore, no association of either oral NK cell or oral ILC numbers was found with plasma viral load, nor was there any direct evidence of increased apoptosis (data not shown), two trends found in colorectal mucosa (Reeves et al., 2011). However, similar to what we have observed in other mucosal sites, NK cell and ILC function in oral tissues was significantly altered during SIV infection. ILCs had nearly a 3-fold increase in intracellular perforin expression in tonsils, as well as a modest, but significant, upregulation of perforin in OLN (Figure 5A). NK cells similarly upregulated intracellular perforin in tonsil and upon stimulation, both NK cells and ILCs from SIV-infected animals upregulated CD107a, a surrogate marker of degranulation and cytotoxicity (Figures 5A,B). CD107a upregulation was, however, only significant in ILCs where the increase was particularly robust (median frequency CD107a expression, naïve −4.5%, SIV-infected −25%). Furthermore, as we have previously found for colorectal tissues, SIV infection induced a loss of IL-17 production in lieu of upregulated TNF-α and IFN-γ production by ILCs. Interestingly, both cell populations produced similar levels of MIP-1β regardless of infection status. Using multiparametric analysis we found very little difference in the multi-function profiles of NKG2A^+^ NK cells (Figure 5C). However, NKp44^+^ did exhibit a significant increase (*p* = 0.01) in overlapping functions, most notably the increase in TNF-α^+^IFN-γ^+^MIP-1β^+^ cells with and without the SIV-induced upregulation of degranulation marker, CD107a.

## Discussion

Here we report one of the first comprehensive quantitative and functional descriptions of both classical NK cells and ILCs in various compartments of the oropharyngeal mucosa. Both cell types were found at relatively low frequencies in rhesus macaque oral tissues, but displayed highly diverse functional profiles. Furthermore, we report a SIV-induced expansion and functional skewing of ILCs that is in stark contrast to the loss of ILCs found in the GI mucosa during chronic lentivirus infection.

In comparison to other routes of mucosal transmission, oral HIV infection is much less common, but the mechanisms of resistance are unclear. High concentrations of salivary enzymes and a thicker epithelial layer provide a better physical barrier, but the contributions of innate immune defenses are poorly understood. HIV/SIV both replicate in macrophages and CD4^+^ T cells in the oral mucosa, regardless of the route of infection (Cutler and Jotwani, 2006; Moutsopoulos et al., 2006; Lu and Jacobson, 2007) and virus replication seems to activate toll-like receptors (TLR) that are found at high densities throughout the oral cavity (Challacombe and Naglik, 2006). Virus ligation of TLRs in DCs and macrophages could indirectly activate and/or recruit NK cells to local foci of infection and TLR upregulation does seem to correlate with NK cell infiltration following experimental oral SIV infection (Milush et al., 2007; Durudas et al., 2011). Active SIV replication in the oral mucosa also induces upregulation of IFN-γ and granzymes, as well as other NK cell biomarkers such as CD16, NKG2C, and relative KIR expression (George et al., 2009), all indicative of a robust NK cell response. These data fit in well with the activation and increased anti-viral functions of NK cells and ILCs we report here for chronically infected animals (Figure 5). Innate responses likely contribute to blocking many potential infections at the portal of entry, and indeed empirical evidence suggests robust NK cell responses strongly correlate with protection against MTCT (Tiemessen et al., 2009; Paximadis et al., 2011). Therefore, the data herein could offer insights into what responses might be necessary to induce protection, and if delineated more clearly, could be harnessed in the context of a vaccine or immunotherapeutic.

Interestingly, the massive ILC and NK cell depletion observed in the GI tract during chronic lentiviral disease is not recapitulated in the oral mucosa. Since ILCs and NK cells are not directly infected, this tissue-specific phenomenon is most likely dependent on changes in upstream regulatory factors. Indeed, we have shown previously that in the GI tract loss is dependent, at least in part, on upregulation of indoleamine deoxygenase {Reeves et al., 2011#6008}. Because a loss was not observed in the oral mucosa, it would be tempting to speculate the same HIV/SIV-induced changes in inflammatory mediators are not present in the oral mucosa. While tissue samples were not available in sufficient quantities for those analyses in this study, future investigations should include a direct comparison of the two tissues. A better understanding of the mechanisms of maintenance in the oral mucosa could point to novel strategies to preserve or restore ILC and NK cell function in the GI tract.

Breakdown of the oral mucosal epithelial barrier in HIV disease is common due to increased inflammation and is thought to contribute to the plethora of opportunistic infections found in progressive disease. Indeed chronic HIV disease is commonly accompanied by oral warts, oral hairy leukoplakia, oral cancers, and fungal infections such as candidiasis (Leigh et al., 2004). The anti-tumor capabilities of NK cells are well-documented (Caligiuri, 2008), but it is unclear if ILCs also possess these functions, despite their upregulation of cytolytic markers (Figure 5). Classical NK cells seem to have little inhibitory effects on fungi such as candida (de Repentigny et al., 2004), but ILCs could harbor anti-fungal properties due to secretion of IL-17 and IL-22. ILC production of IL-17 and IL-22 are also likely to contribute to epithelial homeostasis and the expansion we observe in SIV-infected animals (Figure 4) could be interpreted as a compensatory mechanism in the face of significant mucosal barrier disruption. Alteration of the NK cell and ILC repertoires in the oral mucosae are also likely to contribute to changes in the microbiome that are common in lentivirus infections and could have significant ramifications for health and disease (Brenchley et al., 2006; Lu and Jacobson, 2007; Satoh-Takayama et al., 2008; Ilan, 2009; Sanos et al., 2009). Finally, we must also consider an alternate hypothesis whereby a virus or opportunistic infection-induced activation of NK cells and ILCs might actually lead to increased non-specific cell killing, inflammation, and apoptosis, thus contributing to the breakdown of the oral mucosal barrier. Regardless, these data provide new and interesting insight into the complex role(s) NK cells and ILCs play in the oral mucosa and warrant further study.

### Conflict of interest statement

The authors declare that the research was conducted in the absence of any commercial or financial relationships that could be construed as a potential conflict of interest.
